# Exploring provider and parental perceptions to influenza vaccination in the inpatient setting

**DOI:** 10.1111/irv.12482

**Published:** 2017-12-14

**Authors:** Suchitra Rao, Victoria Fischman, Angela Moss, Sonja I. Ziniel, Michelle R. Torok, Heidi McNeely, Daniel Hyman, Karen M. Wilson, Amanda F. Dempsey

**Affiliations:** ^1^ Division of Infectious Diseases, Hospital Medicine and Epidemiology Department of Pediatrics University of Colorado School of Medicine and Children's Hospital Colorado Aurora CO USA; ^2^ Tufts University School of Medicine Boston MA USA; ^3^ Adult and Child Center for Health Outcomes Research and Delivery Science University of Colorado School of Medicine Aurora CO USA; ^4^ Division of Hospital Medicine Department of Pediatrics University of Colorado School of Medicine and Children's Hospital Colorado Aurora CO USA; ^5^ Department of Pediatrics Adult and Child Center for Health Outcomes Research and Delivery Science University of Colorado School of Medicine Aurora CO USA; ^6^ Division of Nursing Children's Hospital Colorado Aurora CO USA; ^7^ Division of Hospital Medicine and Quality and Patient Safety Department of Pediatrics University of Colorado School of Medicine and Children's Hospital Colorado Aurora CO USA; ^8^ Department of Pediatrics Icahn School of Medicine at Mount Sinai and Kravis Children's Hospital New York NY USA

**Keywords:** barriers, influenza vaccination, inpatient vaccination

## Abstract

**Background:**

Hospitalization provides an ideal opportunity for immunization, but few studies have explored provider and parental attitudes toward pediatric inpatient vaccination against influenza.

**Objectives:**

The objectives were to determine provider and caregiver attitudes and explore potential barriers to inpatient influenza vaccination.

**Methods:**

We developed and distributed two surveys to parents/caregivers as well as providers of general pediatric inpatients at Children's Hospital Colorado between October 2014 and March 2015 assessing attitudes toward influenza and inpatient influenza vaccination. We analyzed the Likert scale responses using univariate analyses and multiple logistic regression to assess associations between responses and vaccination status.

**Results:**

The overall response rate was 95% and 58% for parents and providers, respectively. Parents of hospitalized children who agreed that flu vaccines are safe (adjusted OR 2.50 [95%CI 1.76‐3.58]), and that the influenza vaccine is needed every year had higher odds of having a vaccinated child (adjusted OR 3.30 [95%CI 2.30‐4.81]). Most providers (91%) agree that influenza vaccination is an important priority among inpatients, but believe that parental misconceptions and their reluctance for inpatient vaccination are the most important barriers to influenza vaccination. Providers forgetting to ask about vaccination status and order the vaccine are the next most commonly identified barriers. In contrast, most parents surveyed had favorable attitudes toward inpatient influenza vaccination and disagreed that their child was too sick to receive the vaccine during hospitalization.

**Abbreviations**
CHCO: Children's Hospital Colorado
ED: Emergency department
PCP: primary care provider
QI: Quality Improvement

## 1 INTRODUCTION

Despite universal influenza vaccination policies in the United States to vaccinate everyone aged 6 months and older, national influenza vaccination rates among children remain low. During the 2015‐2016 season, vaccination rates were estimated to be 59.3% among children 6 months to 17 years.^1^ Studies suggest immunization rates among pediatric inpatients are lower than the general population.^2^, ^3^ However, influenza vaccination can be performed even in the setting of acute illness, and hospitalizations provide ideal opportunities for immunization, particularly for individuals with underlying conditions increasing their risk of influenza‐related complications.^4^, ^5^, ^6^

In addition to common reasons for underimmunization described in primary care, such as lack of knowledge, fear or mistrust, and poor healthcare access,^7^, ^8^ hospitalized children may experience additional barriers to immunization. Parents and providers may be reluctant to accept inpatient vaccination due to illness or fever, or believe that vaccination should occur in the primary care setting.^2^ Furthermore, children with chronic health conditions may be hospitalized when their immunizations are due. The objectives of our project were to determine provider and parental attitudes of inpatient influenza vaccination and explore potential barriers to inpatient influenza vaccination, which helped inform initiatives to increase influenza vaccination rates during hospitalization.

## 2 METHODS

### 2.1 Population

Parent/primary caregiver (henceforth called “parent”) survey: Parents of all patients 6 months of age and older (no upper age limit) admitted to three inpatient medical teams at Children's Hospital Colorado between October 2014 and March 2015 were eligible for survey participation. Questionnaires were distributed to parents regarding attitudes toward influenza immunization during hospitalization. Parents were approached after approval from the primary medical or nursing team.

### 2.2 Provider survey

In addition, we surveyed staff working on the medical inpatient units who are authorized to order vaccine (including nursing staff, nurse practitioners, residents, and attendings of the medical inpatient units) regarding perceived barriers to influenza vaccination, and their likelihood of ordering influenza vaccination.

### 2.3 Survey design and procedures

We developed the surveys with input from local immunization experts and survey methodologists, review of the literature, pre‐tested them with inpatient families and hospitalist providers, and modified questions based on their feedback. Response choices were based on a 5‐point Likert scale, with most responses ranging from strongly disagree to strongly agree (Appendix S1, online). Parents were approached by the study team and a paper survey was completed during their child's hospitalization. A questionnaire was sent to inpatient physicians, residents, nurses, physician assistants, and nurse practitioners via e‐mail with three additional reminders, regarding attitudes toward influenza immunization during hospitalization. Influenza vaccination status for the current season upon admission was determined by parental report, with verification in the Colorado Immunization Information System (CIIS) when available. The provider survey was administered through email using Research Electronic Data Capture.^9^

#### 2.3.1 Data analysis

Data were analyzed separately for providers and parents. Duplicate responses from parents of a child who had more than one admission were removed prior to analysis. Descriptive statistics were generated for all survey questions. Likert scale responses were dichotomized into agree (strongly agree/agree) or do not agree (neither agree nor disagree/disagree/strongly disagree) based on distribution of responses. Chi‐square tests explored associations between baseline vaccination status and parental perceptions of influenza illness and vaccine. Parental responses with a significance level of <0.2 in the bivariate analysis were modeled separately using multiple logistic regression. Odds ratios and 95% confidence intervals (CI) were estimated, and results with *P*‐values <.05 were considered statistically significant. Bivariate analyses were conducted comparing responses by provider type (nurse, trainee, physician). Analyses were performed using SAS software, version 9.4 (SAS Institute, Cary, NC, USA).

### 2.4 Approvals

The survey was conducted as a component of a Quality Improvement initiative with approval by the Organizational Research Risk and Quality Improvement Review Panel (ORRQIRP) (QI# 1403‐8).

## 3 RESULTS

### 3.1 Parent surveys

The overall response rate was 1001/1053 (95%): Of these, 392 (37%) of children received the current influenza vaccine at the time of admission. The median age of the children of the survey respondents was 3.9 years (IQR 1.6‐10), 53% had government insurance, 69.8% were white, 54% were male, and 43.6% had a high‐risk medical condition. Of the 55% (546/1001) of parents stating that they usually get annual influenza vaccination for their child, 502 of 546 (92%) would agree to inpatient influenza vaccination if eligible. Among the 45% (455/1001) of parents who reported they not routinely seek influenza vaccination for their child, 84/229 (37%) would agree to inpatient influenza vaccination if eligible. Parents of children already vaccinated at admission were more likely to agree to hypothetical inpatient vaccination if eligible (71%) compared with unvaccinated children (53%), *P* = .0025.

**Figure 1:**
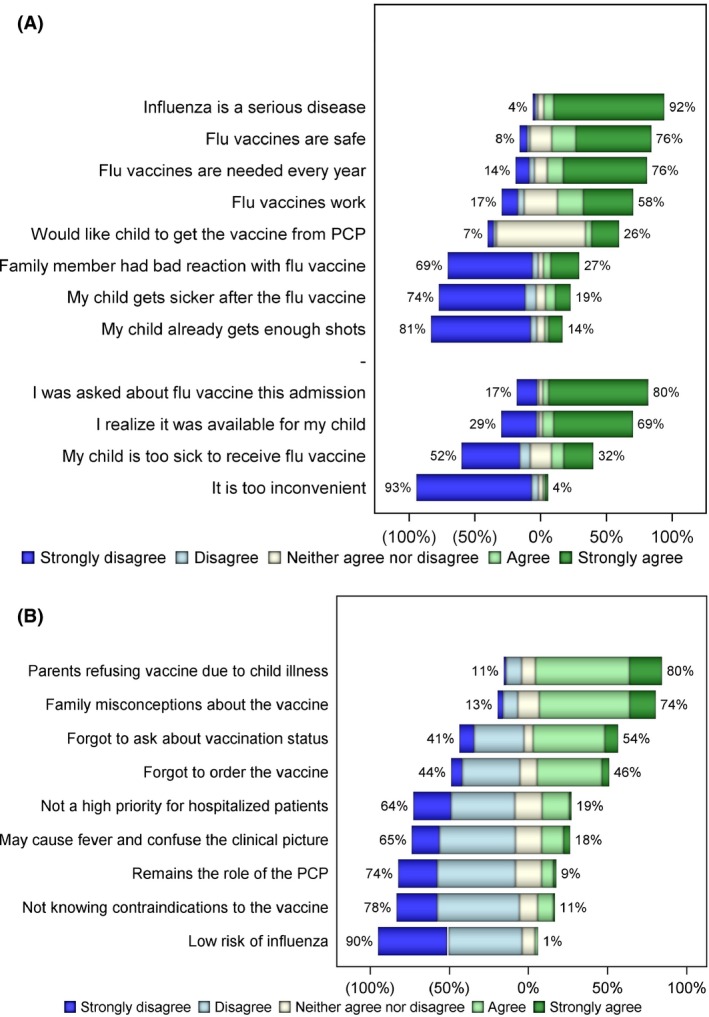
Bar graph showing responses to questions regarding barriers to influenza vaccination among (A) parents and (B) providers of children hospitalized on the medical inpatient units at Children's Hospital Colorado, 2014‐2015. Figure demonstrates percentage of parent and provider responses to Likert scale responses from survey questions. Percentages represent the grouping of “strongly disagree and disagree” or “agree and strongly agree.” Parents understood the severity of influenza and tended to have a favorable response to vaccination in the inpatient setting. Providers believed the strongest barriers to inpatient vaccination were parental refusal and family misconceptions of the vaccine, followed by forgetting to ask about and order influenza vaccine. PCP, primary care provider

Parental attitudes to influenza vaccination are shown in Figure 1A. Parents agreed that influenza is serious (92%), that influenza vaccines work (58%), are safe (76%) and needed each year (76%). Bivariate and multivariate analyses of children vaccinated versus unvaccinated at admission are shown in Table 1. Parents of hospitalized children who agreed that flu vaccines are safe, and that influenza vaccination is required every year, had higher odds of having a vaccinated child. Parents who agreed their child gets enough shots were less likely to have a vaccinated child.

**Table 1:** Bivariate and multiple logistic regression models of parental survey responses evaluating barriers to influenza vaccination by child's vaccination status^a^

Independent variable	Unadjusted logistic models	Multiple logistic models	
	Unadjusted odds ratio (95% CI)	Adjusted odds ratio (95% CI)	*P* value
My child is too sick to receive the flu vaccine	1.37 (1.03, 1.82)	1.35 (1.00, 1.82)	.051
Flu vaccines work	1.25 (0.95, 1.64)	1.32 (0.993, 1.75)	.056
Flu vaccines are safe	2.59 (1.85, 3.66)	2.50 (1.76, 3.58)	<.0001
I would like my child to get the vaccine at the pediatrician's office	1.22 (0.90, 1.66)	1.16 (0.84, 1.61)	.356
A family member has had a bad experience with flu vaccines	0.79 (0.58, 1.07)	0.81 (0.59, 1.10)	.177
Flu vaccines are needed every year	3.25 (2.28, 4.69)	3.30 (2.30, 4.81)	<.0001
My child already gets enough shots	0.43 (0.27, 0.65)	0.43 (0.27, 0.66)	.0002

a For all models, the dependent variable was the vaccination status of the patient (vaccinated/not vaccinated). Parental survey responses with a significance level of <0.2 in the bivariable analysis were modeled independently of each other using logistic regression. Unadjusted logistic regression was used to assess the unadjusted association between each independent variable and vaccination status. Multiple logistic regression was used to adjust the association for baseline imbalances of insurance status, high‐risk status, and age. The following table presents the results of the unadjusted and adjusted odds ratios (OR) and the corresponding 95% CI values. For each independent variable, the referent group was the “do not agree” response.

### 3.2 Provider survey results

The overall response rate was 195 of 339 (58%) for providers. Most provider surveys were completed by nurses (40%), followed by residents/fellows (35%) and attending physicians (13%). Non‐responders comprised nurses (48%), residents/fellows (23%), and only 2% were attending physicians. There was no significant difference in response rates between nurses, attending physicians, and trainees.

Provider report of parental refusal due to child illness (80%) and family misconceptions of the vaccine (74%) were the most common barriers to vaccination perceived by providers. In addition, 54% and 46% of providers forgot to ask about influenza vaccination status or order the vaccine, respectively (Figure 1B).

Most providers agreed that vaccination in the inpatient setting was important/very important (91%), and ordered vaccine often/sometimes (87%). When asked about interventions to increase influenza vaccination rates in the inpatient setting, 73% of providers agreed that personal reminders may be helpful to increase vaccination rates; however, only 48% believed that provider education may be helpful to increase vaccination rates.

## 4 DISCUSSION

The major finding of this study is the dichotomy between provider and parental attitudes toward inpatient influenza vaccination. We found that most providers agree that influenza vaccination is an important priority among inpatients, but find parental misconceptions and their reluctance for inpatient vaccination are important barriers to influenza vaccination. Providers forgetting to ask about vaccination status and order the vaccine are the next most commonly identified barriers. In contrast, more than half of parents surveyed had favorable attitudes toward inpatient influenza vaccination and disagreed that their child was too sick to receive the vaccine during hospitalization.

Our study demonstrates that parental beliefs and attitudes contribute to suboptimal influenza vaccination rates, similar to other studies, but suggests that other factors exist for our inpatient populations. We found that parents of children unvaccinated at admission were more likely to disagree that vaccines are safe, that they work well to prevent influenza, and that annual influenza vaccination is required, than the parents of vaccinated children. Other studies identified similar safety concerns and identified beliefs about the potential severity and susceptibility to influenza as important barriers.^10^, ^11^, ^12^ However, over half the parents of unvaccinated children surveyed in our study were willing to entertain inpatient influenza vaccination. Therefore, while attitudes and beliefs play a role, the favorable attitudes among our parents surveyed raise possibilities for other potential barriers such as lack of access. This highlights a significant opportunity for vaccination during hospitalization to help overcome such barriers, whereby providers can target eligible children, including some of our more vulnerable populations.

Several limitations of the study exist. First, the study was conducted in the setting of a quality improvement project at a single center, so its application to other settings is limited. Second, the sample may have been biased toward parents or providers with a more favorable attitude toward vaccination. Next, we did not explore the relationship between provider attitudes and vaccination status during admission, or the influence of provider attitudes on parental perceptions. Finally, the accuracy of vaccination status may be restricted as it was based on parental recall, but we did verify vaccination status in the state vaccination registry.

In conclusion, our findings suggested that parents may be more open to inpatient influenza vaccination than providers perceive. Provider misconceptions about parental attitudes may pose a more significant barrier to influenza vaccination than the actual parental attitudes themselves. Our findings serve as a reminder that providers should discuss inpatient influenza vaccination with parents, and suggest that interventions that target providers such as education regarding parental beliefs and reminders may be effective strategies to increase influenza vaccination rates among pediatric inpatients.

## CONFLICT OF INTEREST

Amanda Dempsey has provided consulting services to Merck, Pfizer, and Sanofi Pasteur but does not receive any research funding from these entities. All other authors have no conflict of interests to report.
