# Supplementary material for: Exploring provider and parental perceptions to influenza vaccination in the inpatient setting
Source: Influenza Other Respir Viruses. 2017 Dec 14;12(3):416–20. doi: 10.1111/irv.12482 (PMC5907817; doi:10.1111/irv.12482)
Supplement: Supplementary file 1 [file IRV-12-416-s001.docx]

**Appendix 1, online. Questionnaires**

**Questionnaire for providers**

Which of the following do you perceive as potential barriers to ordering influenza vaccination for inpatients?

|  | Strongly disagree | Disagree | Neither agree nor disagree | Agree | Strongly agree |
| --- | --- | --- | --- | --- | --- |
| Forget to ask about vaccination status | 1 | 2 | 3 | 4 | 5 |
| Forget to order the vaccine | 1 | 2 | 3 | 4 | 5 |
| Not a high priority for hospitalized patients | 1 | 2 | 3 | 4 | 5 |
| May cause fever and confuse clinical picture | 1 | 2 | 3 | 4 | 5 |
| Remains the role of the PCP, not in the hospital setting | 1 | 2 | 3 | 4 | 5 |
| Not knowing contraindications to vaccine | 1 | 2 | 3 | 4 | 5 |
| Parents refusing vaccine because child is too ill | 1 | 2 | 3 | 4 | 5 |
| Low risk of influenza | 1 | 2 | 3 | 4 | 5 |
| Family misconceptions about the vaccine | 1 | 2 | 3 | 4 | 5 |

| Please answer the following questions regarding influenza vaccination ordering: | | | | |  |
| --- | --- | --- | --- | --- | --- |
| How important is vaccination against influenza for inpatients? | Unimportant | Of little importance | Moderately Important | Important | Very important |
| Do you order influenza vaccine for inpatients? | Never | Rarely | Sometimes | Often | Always |
| Do you use the flu  Best Practice Alert? | Never | Rarely | Sometimes | Often | Always |
| Do you use the Influenza Vaccine Dashboard? | Never | Rarely | Sometimes | Often | Always |
| Do you think that personal reminders will help increase your vaccine ordering? | Not much | Little | Somewhat | Much | A great deal |
| Would education about the flu vaccine help increase your vaccine ordering? | Not much | Little | Somewhat | Much | A great deal |

**Questionnaire for parents**

Has your child been vaccinated against influenza this season? Yes no N/A

Do you get your child vaccinated against influenza every year? Yes no N/A

Would you agree to a flu vaccine for your child during the hospitalization if he/she was not yet immunized? Yes no

What are your thoughts regarding the following statements?

|  | Strongly disagree | Disagree | Neither agree nor disagree | Agree | Strongly agree |
| --- | --- | --- | --- | --- | --- |
| My child is too sick to receive the flu vaccine | Strongly disagree | Disagree | Neither agree nor disagree | Agree | Strongly agree |
| Flu vaccines work well to prevent against flu | Strongly disagree | Disagree | Neither agree nor disagree | Agree | Strongly agree |
| My child gets more sick after the influenza vaccine | Strongly disagree | Disagree | Neither agree nor disagree | Agree | Strongly agree |
| Flu vaccines are safe | Strongly disagree | Disagree | Neither agree nor disagree | Agree | Strongly agree |
| I would like my child to get the vaccine at the pediatrician’s office | Strongly disagree | Disagree | Neither agree nor disagree | Agree | Strongly agree |
| I realize that the flu vaccine was available for my child | Strongly disagree | Disagree | Neither agree nor disagree | Agree | Strongly agree |
| I was asked about the flu vaccine this admission | Strongly disagree | Disagree | Neither agree nor disagree | Agree | Strongly agree |
| It is too inconvenient | Strongly disagree | Disagree | Neither agree nor disagree | Agree | Strongly agree |
| Influenza (flu) is a serious disease that can make people sick, even die | Strongly disagree | Disagree | Neither agree nor disagree | Agree | Strongly agree |
| A family member has had a bad experience with flu vaccines before | Strongly disagree | Disagree | Neither agree nor disagree | Agree | Strongly agree |
| My child already gets enough shots | Strongly disagree | Disagree | Neither agree nor disagree | Agree | Strongly agree |
| The flu vaccine is needed every year | Strongly disagree | Disagree | Neither agree nor disagree | Agree | Strongly agree |
| My child has an egg or vaccine allergy | Strongly disagree | Disagree | Neither agree nor disagree | Agree | Strongly agree |
